# Endoglucanase gene of M42 aminopeptidase/endoglucanase family from thermophilic *Bacillus* sp. PW1 and PW2 isolated from Tattapani hot spring, Himachal Pradesh, India

**DOI:** 10.1186/s43141-019-0001-8

**Published:** 2019-10-02

**Authors:** Divyanshi Sharma, Parul Sharma, Kamal Dev, Anuradha Sourirajan

**Affiliations:** 1 Faculty of Applied Sciences and Biotechnology, Shoolini University, PO Box 9, Anand Campus, The Mall, Solan, Himachal Pradesh 173212 India; 2 Department of Food Science and Nutrition, University of Minnesota-Twin Cities, St. Paul, MN USA

**Keywords:** Thermophilic, Tattapani, *Bacillus*, Endoglucanase, M42 aminopeptidase/endoglucanase family, Dual enzyme

## Abstract

**Background:**

Thermostable cellulases are in constant demand for several biotechnological applications. Two thermophilic bacterial strains PW1 and PW2 isolated from Tattapani hot spring were found to have cellulolytic activity. Subsequently, PW1 and PW2 were identified and mined for genes encoding cellulase activity.

**Results:**

Sequencing of the 16S rDNA of PW1 and PW2 identified them as *Bacillus* sp. PW1 (Acc no. KU711837) and *Bacillus* sp. PW2 (Acc no. KU711838), respectively, which clustered in the clades containing thermophilic members of *Bacillus* sp. and *Geobacillus* species. Phylogenetic analysis revealed that despite the morphological and sequence identities, *Bacillus* sp*.* PW1 and *Bacillus* sp. PW2 are different at the genetic level. The cellulase genes (~ 1.1 kb) of the two bacterial strains were amplified using primers designed against related thermophilic cellulases. Sequencing of the cellulase gene amplicons of PW1 and PW2 revealed that they encode proteins of 280 and 206 amino acid residues, respectively. Sequence and domain analysis of the protein products of PW1 and PW2 revealed that they belong to M42 family of aminopeptidase/endoglucanase. The PW2 endoglucanase coding sequence was submitted to Genbank under accession no. MH049504. The structures of putative endoglucanases of PW1 and PW2 were generated using 1VHE.A as template, which showed the presence of vast proportion of random coils. Molecular docking of the modeled endoglucanase proteins with various substrates and products of cellulases showed that carboxymethyl cellulose and maltose exhibit the highest binding affinity, while xylan and glucose the least.

**Conclusions:**

The two thermophilic bacteria PW1 and PW2 and their endoglucanase gene can be further utilized for recombinant production of thermostable cellulases for their application in industries.

**Electronic supplementary material:**

The online version of this article (10.1186/s43141-019-0001-8) contains supplementary material, which is available to authorized users.

## Background

Degradation of cellulose by cellulolytic enzymes is mediated by differential cleavage of the glycosidic bonds linking the monomers/chains [1]. Several bacteria produce a group of enzymes known as cellulases, which act in a concerted manner to hydrolyze the β-1, 4-d-glycosidic bonds within the cellulose molecules [2]. Cellulases comprise three types of activities: endoglucanases or carboxymethylcellulases (CMCases; endo-β-1, 4-glucanases; EC 3.2.1.4), exoglucanases or cellobiohydrolases (CBHases; exo-β-1,4-glucanase; EC 3.2.1.91), and β-glucosidases (β-d-glucoside glucohydrolase; EC 3.2.1.21) [3–5]. Endoglucanases hydrolyze internal β-1, 4-D-glycosidic bonds in cellulose [6]. As a result, the length of the polymer decreases rapidly, and the concentration of reducing sugar increases slowly. Exoglucanases hydrolyze cellulose by detaching the cellobiose units from the non-reducing ends of cellulose [2, 7]. Cellulases are produced by several organisms, including bacteria and fungi [8, 9]. Thermophilic microbes are utilized for their cellulolytic potential due to fast growth rates at high temperatures, presence of complex multienzymes, and their occurrence in diverse environmental niches. There are a few thermophilic bacteria that are potent producers of cellulases, including *Bacillus subtilis*, *Geobacillus pallidus*, *Bacillus licheniformis*, *Bacillus pumilis*, *Aneurinibacillus thermoaerophilus*, and *Clostridium thermocellum* [10]. Thermophilic enzymes are ideal biocatalysts for the present day biotechnology because of their thermo-stability [11] and better yields under intense operational conditions [12].

Thermostable cellulases are gaining wide industrial and biotechnological significance due to their amenable use in harsh industrial processes like paper and textile production [13–17]. Moreover, the use of elevated temperatures in bioconversions minimizes the risk of contamination by common mesophiles and enhances the bioavailability and solubility of organic compounds [18]. Other values of elevated process temperatures include higher reaction rates due to a decline in viscosity and an increase in the diffusion coefficient of substrates and higher process yield due to enhanced solubility of substrates and products and favorable equilibrium displacement in endothermic reactions [19, 20]. Such cellulase enzymes can also be used as models for understanding thermo-stability and thermo-activity, which is beneficial for protein engineering. The hot springs of north-west Himalayas harbor a plethora of thermophilic microbes [21]. One such hot spring is located in Tattapani, Himachal Pradesh, India [21]. Diverse thermophiles were isolated from Tattapani hot spring possessing a variety of hydrolytic enzyme activity [21]. Previously, we reported the features of extracellular thermophilic cellulase produced by *Geobacillus* sp. of Tattapani hot spring [21]. In view of the importance of thermophilic cellulases for biotechnological applications, the present study was undertaken to screen thermophiles for cellulase activity. Two thermophilic bacterial strains PW1 and PW2 of Tattapani hot spring were screened and found to have cellulolytic activity. Towards overexpression of the thermophilic cellulase in recombinant system, genes encoding the cellulase activity of the two bacterial strains were mined and characterized by in silico studies.

## Methods

### Strains used in the study

Two thermophilic bacterial strains PW1 and PW2 were isolated from water samples of hot spring (water temperature ~ 70 °C) located in Tattapani, district Mandi, Himachal Pradesh, India [21]. The bacterial strains were cultured in nutrient broth (NB; Himedia Labs, India) and stored on nutrient agar at 4 °C and as 50% glycerol stocks at − 80 °C.

### Biochemical analyses of the bacterial isolates

Biochemical tests such as catalase, urease, nitrate reduction, and oxidase tests were carried out to study the biochemical characteristics of thermophilic isolates PW1 and PW2 [22].

### Optimization of physical parameters for growth of PW1 and PW2

Bacterial isolates were grown in nutrient broth adjusted to different pH ranging from 4 to 10, and incubated at 60 °C for 24 h with shaking at 200 rpm. Effect of temperature was measured by culturing the bacterial isolates in nutrient broth at different incubation temperatures (30–80 °C) for 24 h. Cell density was determined by measuring absorbance at 600 nm. The growth profile at higher than 80 °C could not be studied due to evaporation of media at high temperature.

### Screening of chemical parameters for growth of PW1 and PW2

Bacterial isolates were grown in minimal salt medium (M9) containing NH_4_Cl as nitrogen source supplemented with 1% of different carbon sources such as glucose, starch, sucrose, fructose, lactose, raffinose, galactose, glycerol, and sorbitol. Minimal salt medium (M9) containing glucose as carbon source complemented with 0.25% of different nitrogen sources such as yeast extract, peptone, tryptone, beef extract, casein hydrolysate, urea, and NH_4_Cl was used to study the role of nitrogen source on growth. The bacterial cultures of PW1 and PW2 were incubated at 60 °C with shaking at 200 rpm for 24 h. The effect on growth of PW1 and PW2 was studied by measuring cell density at 600 nm. Due to evaporation of media at high temperature, we could not study growth profile at temperatures higher than 80 °C.

### Screening and estimation of cellulase activity of thermophilic bacterial isolates PW1 and PW2

The thermophilic bacterial isolates (PW1 and PW2) were grown at 60 °C, and an equal number of cells was spotted on NB agar medium supplemented with 1% carboxymethyl cellulose (CMC) to screen for cellulase activity and incubated at 60 °C for 24 h. The CMC agar plate was stained with Gram’s iodine solution (1%). The appearance of a clear zone around the bacterial growth indicated the utilization of CMC, thus indicative of cellulase activity. Cellulase activity was measured by the DNS method [23], through the determination of the amount of reducing sugars liberated from carboxymethyl cellulose (CMC) at 540 nm. Twenty micrograms of total protein as crude cell-free enzyme was added to 0.2 ml of 1% CMC. The reaction mixture was incubated at 60 °C for 30 min, and the reaction was stopped by the addition of 2.0 ml DNS reagent. Enzyme activity was calculated. One unit of enzyme activity is defined as the amount of enzyme liberating 1 μg of glucose. Enzyme blank and substrate blank were performed in the assays with only added enzyme source or substrate, respectively.

### Molecular identification of cellulolytic thermophilic bacterial isolates PW1 and PW2 by 16S rDNA sequencing

The thermophilic bacterial isolates were cultured at 60 °C to an *A*_600_ of ~ 1.0, and the cells were harvested by centrifugation at 12,000 rpm for 10 min. Genomic DNA from each of the bacterial cell pellet was isolated as described [24]. For identification of thermophilic bacterial isolates, 100 ng of genomic DNA was subjected to PCR amplification of 16S rDNA gene using 27F and 1492R primers (Additional file 1: Table S1) [25]. The PCR thermal cycling conditions were as follows: initial denaturation at 94 °C for 2 min followed by 35 cycles of denaturation (94 °C, 30 s), annealing (45 °C, 30 s), and extension (72 °C, 2 min), with a final extension of 10 min at 72 °C. The PCR products were resolved on 1% agarose gel. The gel-purified PCR products of 16S rDNA gene were sequenced on both strands using the primers 27F and 1492R at Eurofins, Bangalore, India (https://www.eurofins.com). The nucleotide sequences were manually analyzed, overlapping sequences were removed, and the complete 16S rDNA sequence for both the bacterial strains was generated. The nucleotide sequences were analyzed by BLAST (blastn) search and compared against bacterial 16S rDNA sequences available in the Genbank database [26]. The phylogenetic tree was constructed using MEGA4 (http://www.megasoftware.net) [27]. The nucleotide sequences were submitted in the GenBank database (https://www.ncbi.nlm.nih.gov/genbank/).

### Isolation of gene encoding cellulase from *Bacillus* sp. PW1 and *Bacillus* sp. PW2

To amplify the cellulase gene from the thermophiles PW1 and PW2, gene-specific primers were designed using cellulase gene of *Geobacillus* sp. Y412MC52 [GYMC52-2749]. The nucleotide sequence of the primers used for PCR amplification of cellulase gene is listed in Additional file 1: Table S1. The following PCR conditions were used for amplification of cellulase gene from genomic DNA of *Bacillus* sp. PW1 and *Bacillus* sp. PW2: initial denaturation at 94^ο^C, 2 min, and 35 cycles of the following steps: denaturation at 94^ο^C, 30 s; annealing at 48^ο^C, 30 s; extension at 72^ο^C, 2 min; and final extension at 72^ο^C, 10 min. The amplified PCR products were resolved on 1% agarose gel, visualized under UV gel documentation system (Alpha Innotech, USA), and then purified with a gel extraction kit (Thermo Scientific Inc., USA). The gel-purified PCR products were subjected to sequencing on both the strands using the same primers. The complete cellulase gene sequence for each bacterial isolate was generated manually by removing overlapping sequences. The nucleotide sequence was converted into protein sequence using the Expasy translation tool (https://web.expasy.org/translate)*.* The protein sequence obtained for each bacterial isolate was compared against other known bacterial protein sequences available in the Genbank database using BLAST (BLASTP) search [26]. The phylogenetic tree was constructed by neighbor joining using MEGA4 [27].

### Multiple sequence alignment of predicted PW1 endoglucanase and PW2 endoglucanase protein sequences with their homologs

The amino acid sequences showing more than 90% similarity from BLAST hits against PW1 endoglucanase and PW2 endoglucanase were selected and subjected to sequence alignment using PRALINE software [28] (www.genome.jp/tools/clustalw/).

### Conserved domain analysis and hydropathy plots of predicted PW1 endoglucanase and PW2 endoglucanase

The protein sequences of PW1 endoglucanase and PW2 endoglucanase were subjected to conserved domain analysis using Conserved Domain Database tool of NCBI (http://www.ncbi.nlm.nih.gov/Structure/cdd/). Hydropathy plots were generated using Expasy-Protscale (https://web.expasy.org/protscale/).

### Molecular modeling and in silico docking of PW1 and PW2 endoglucanases with the substrates and reaction products involved in cellulase-mediated catalysis

In order to study enzyme/substrate interaction, homology models of PW1 endoglucanase and PW2 endoglucanase were generated using the Swiss model (https://swissmodel.expasy.org/). The template selected by the Swiss model was aminopeptidase/glucanase homolog of *Bacillus subtilis* strain168(PDB id. 1VHE.A Chain A), which showed 80.71% sequence identity with *Bacillus* sp. PW1 endoglucanase and 76.70% sequence identity with *Bacillus* sp. PW2 endoglucanase, respectively. The ligands selected for docking include CMC, cellulose, xylan, glucose, maltose, and dextrin. The structure of ligands was procured from ChemSpider database (http://www.chemspider.com) in .mol format and then converted to .pdb format using open Babel software (http://openbabel.org/). Docking of the ligands was performed with the surface models of PW1 and PW2 endoglucanases using Hex software (http://hex.loria.fr/). The molecular visualization of structural interactions between PW1 and PW2 endoglucanases and different ligands were studied using Pymol software (https://www.pymol.org/). Amino acids involved in protein-ligand interaction were retrieved using PDB sum software (www.ebi.ac.uk/thornton-srv/databases/pdbsum/Generate.html :).

## Results and discussion

### Thermophilic bacterial isolates PW1 and PW2 possess cellulolytic activity and identified as *Bacillus* sp. PW1 and *Bacillus* sp. PW2

**Fig. 1:**
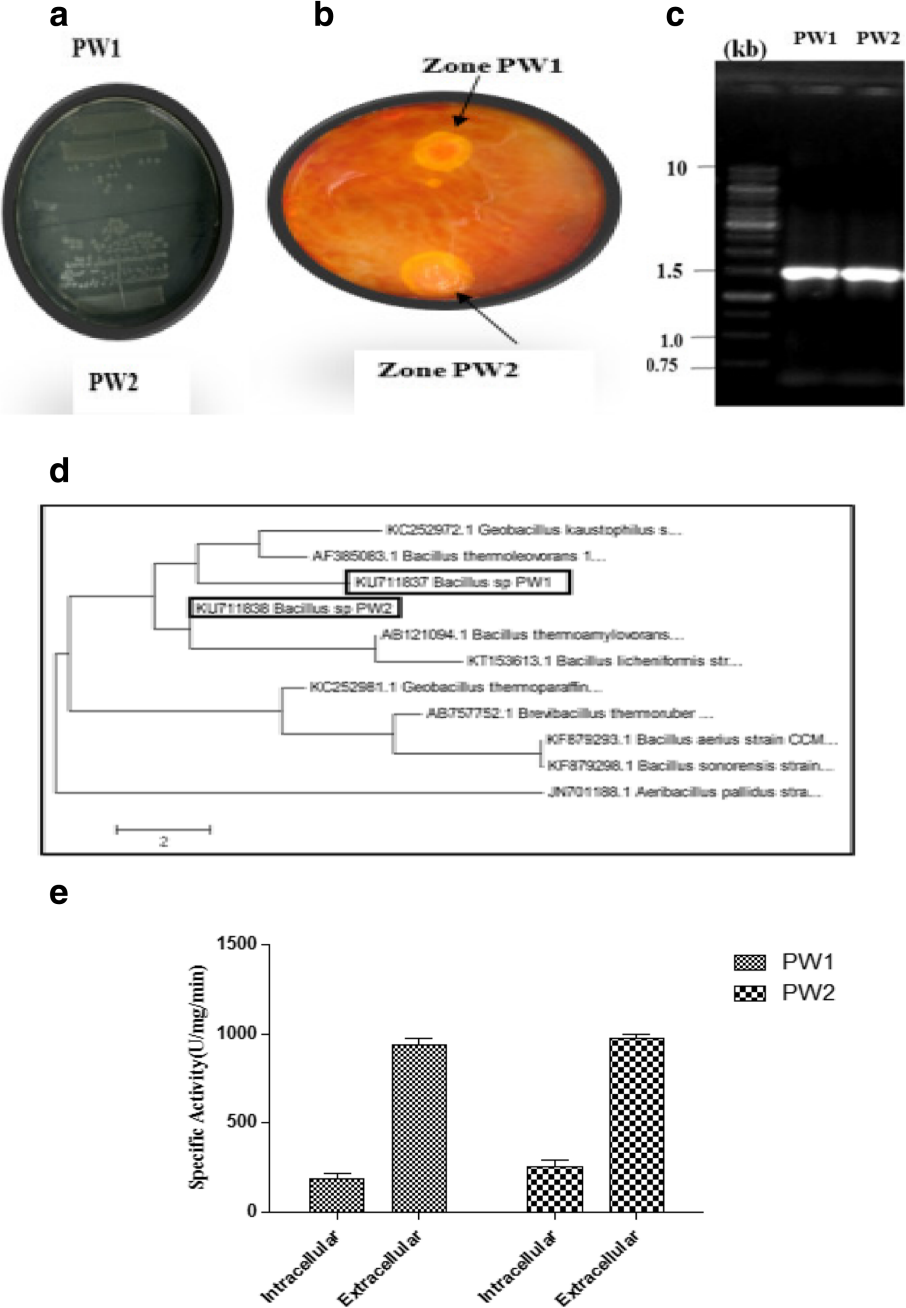
**a** Growth of thermophilic bacterial isolates PW1 and PW2 on nutrient agar at 60 °C. **b** Detection of thermophilic cellulase activity of PW1 and PW2 bacterial isolates. Purified bacterial isolates of PW1 and PW2 were spotted on NA medium supplemented with 1% CMC and incubated at 60 °C for 24 h and flooded with 1% Gram’s iodine. The appearance of clear halo zones around bacterial growth indicates cellulase production by PW1 and PW2. **c** Amplification of 16S rDNA of PW1 and PW2 by PCR. Lanes 1 and 2: PCR products showing 16S rDNA amplification of PW1 and PW2, respectively. The size of 16S rDNA amplicon (~ 1500 bp) is indicated. M size marker (in kb). **d** Phylogenetic analysis of 16S rDNA sequences of *Bacillus* sp. PW1 and *Bacillus* sp. PW2. Dendrogram of PW1 and PW2 with related thermophiles (> 99% homology) is shown. The Jaccard’s coefficient scale for genetic relatedness is indicated below phylogenetic tree. **e** Quantitative cellulase assay of bacterial isolates PW1 and PW2

As shown in Fig. 1b, both PW1 and PW2 bacterial isolates produced clear zones (22 ± 0.1 mm and 20 ± 0.1 mm, respectively) on CMC agar at 60 °C, indicating the presence of cellulase enzyme. Quantification of CMC cellulase activity for both the bacterial species at 60 °C indicated the presence of predominantly extracellular cellulase activity (Fig. 1e). The thermophilic extracellular cellulase activities of PW1 and PW2 were found to be 1015 U/mg/min and 994 U/mg/min, respectively. For molecular identification of PW1 and PW2 isolates, total genomic DNA was isolated and used for amplification of 16S rDNA. An amplicon of 16S rDNA of ~ 1500 bp was observed for both PW1 and PW2 (Fig. 1c). The amplified 16S rDNA products were sequenced on both the strands, and a nucleotide sequence of 1435 bp and 1433 bp was obtained for PW1 and PW2, respectively. Analysis of the sequences by BLAST showed that the cellulolytic isolates PW1 and PW2 belong to *Bacillus* sp. showing 99% similarity to *Bacillus* sp. SP76. The 16S rDNA nucleotide sequences of the two bacterial isolates have been submitted to the NCBI GenBank database under the accession numbers KU711837 (*Bacillus* sp. strain PW1) and KU711838 (*Bacillus* sp. strain PW2), respectively. Phylogenetic analysis of 16S rDNA sequences of PW1 and PW2 with their close homologs revealed that *Bacillus* sp. PW2 clustered in the clade containing members of *Bacillus* and *Geobacillus* genera (Fig. 1d). On the other hand, *Bacillus* sp. PW1 emerged as an independent clade with no related members. It is interesting to note that despite the morphological and sequence identity, *Bacillus* sp. PW1 and *Bacillus* sp. PW2 are different at the genetic level.

### *Bacillus* sp. PW1 and *Bacillus* sp. PW2 exhibit differences in morphological, biochemical, and growth characteristics

At the morphological level, *Bacillus* sp. PW1 appear small rod shaped, forming creamish colonies, while *Bacillus* sp. PW2 are long rod shaped and form whitish colonies (Fig. 1a; Table 1). Both the isolates were positive for Gram’s staining, nitrate utilization, catalase, and oxidase reactions. However, *Bacillus* sp. PW1 was urease negative and glutaminase positive, and *Bacillus* sp. PW2 was urease positive and glutaminase negative (Table 1). Both PW1 and PW2 were found to be strict thermophiles, with no significant growth below 50 °C (Additional file 1: Figure S1). Optimum pH and temperature for growth of both the bacterial isolates were pH 8 and 80 °C, respectively (Additional file 1: Figure S1; Table 1). Among different carbon sources tested, galactose and glucose were the best carbon source for *Bacillus* sp. PW1 and *Bacillus* sp. PW2, respectively (Additional file 1: Figure S2; Table 1). Although all the carbon sources supported the growth of bacteria, sorbitol was found to be a poor carbon source (Additional file 1: Figure S2). Among different nitrogen sources studied, ammonium chloride was the best nitrogen source for the growth of *Bacillus* sp. PW1, while tryptone was the best nitrogen source for the growth of *Bacillus* sp. PW2 (Additional file 1: Figure S2). However, urea and casein hydrolysate were the least effective for the growth of both PW1 and PW2 isolates (Additional file 1: Figure S2). Thus, the two bacterial isolates exhibit differences in enzymatic and growth characteristics, despite being similar at the sequence level.

**Table 1:** Biochemical, morphological, and growth characteristics of thermophilic bacterial isolates PW1 and PW2

Parameter	*Bacillus* sp. PW1	*Bacillus* sp. PW2
Cell morphology	Small rods	Long rods
Pigmentation	Cream	White
Gram’s reaction	+	+
Catalase	*+*	+
Oxidase	*+*	+
Nitrate	*+*	+
Urease	−	+
Glutaminase	+	−
Motility	*+*	+
Optimum pH (range)	8 (6–9)	8 (6–9)
Optimum temperature (range)	80 °C (60–80 °C)	80 °C (60–80 °C)
Carbon source	Galactose	Glucose
Nitrogen source	Ammonium chloride	Tryptone

### *Bacillus* sp. PW1 and *Bacillus* sp. PW2 possess genes encoding endoglucanase of M42 aminopeptidase/endoglucanase family

**Fig. 2:**
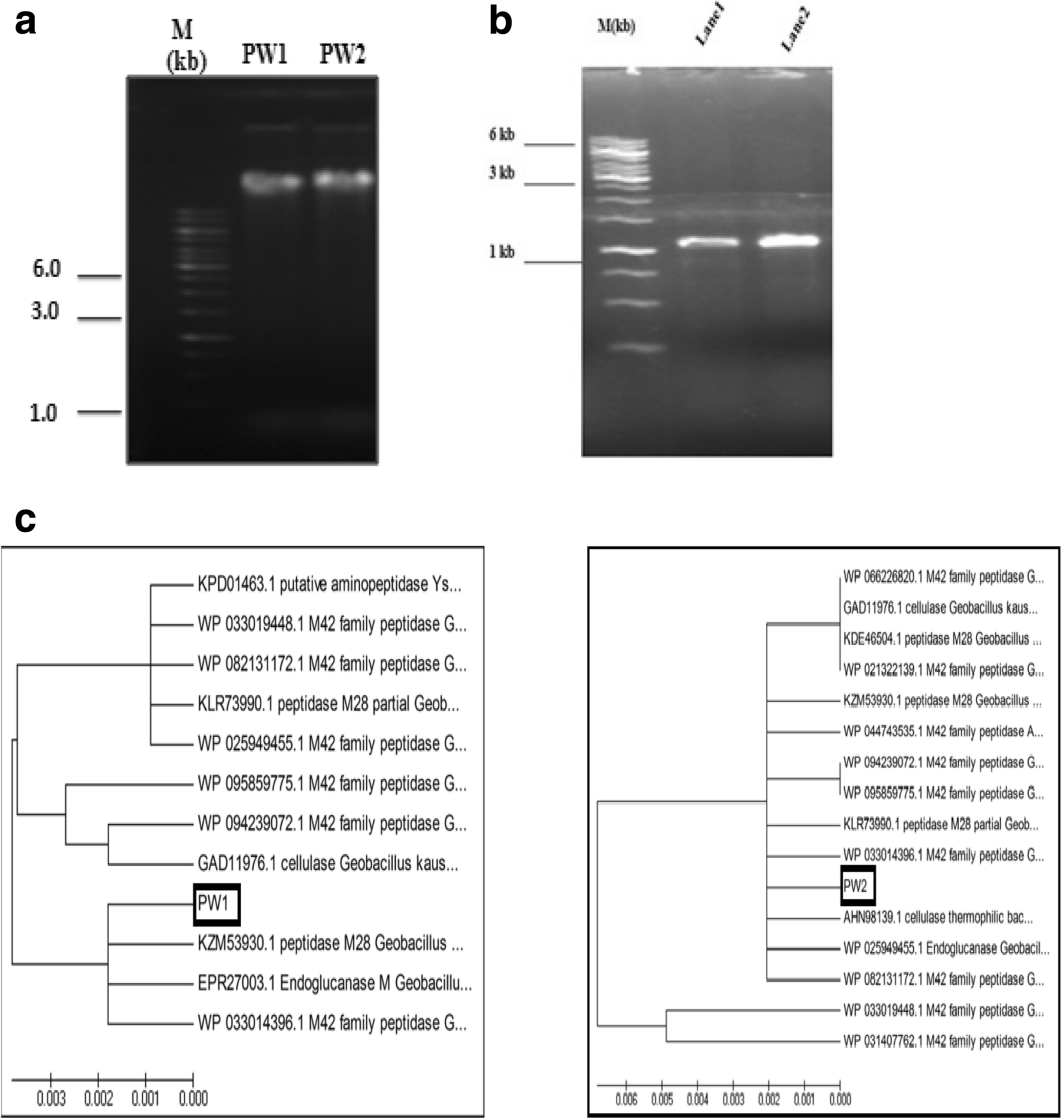
Isolation of genes encoding cellulase from the thermophiles *Bacillus* sp. PW1 and *Bacillus* sp. PW2. **a** Analysis of genomic DNA of PW1 (lane 1) and PW2 (lane 2) by agarose gel electrophoresis. **b** PCR amplification of cellulase gene from genomic DNA of PW1 and PW2. Lanes 1 and 2: PCR products showing amplification of cellulase gene of *Bacillus* sp. PW1 and *Bacillus* sp. PW2 (~ 1.1 kb), respectively. The size marker (M) bands are indicated (kb). **c** Dendrogram showing phylogenetic relationship of PW1 and PW2 endoglucanase protein sequences with their representatives showing > 99% homology. The Jaccard’s coefficient scale for genetic relatedness is indicated below phylogenetic tree

To mine the genes encoding cellulase activity from the two thermophiles, primers were designed based on the available gene sequences from closely related thermophiles. An amplicon of ~ 1.1 kb was observed for both PW1 and PW2 (Fig. 2a, b). The amplified fragments were sequenced on both the strands, and nucleotide sequences obtained were translated into amino acid sequences, yielding ORFs of 280 and 206 amino acids for PW1 and PW2, respectively (Additional file 1: Figure S3 and Figure S4). The translated ORFs were subjected to BLASTp analysis. Interestingly, the ORFs of both *Bacillus* sp. PW1 and *Bacillus* sp. PW2 showed 100% similarity with endoglucanase M of *Geobacillus* sp.WSUCF1 (EPR27003.1), as well as M42 family aminopeptidase of *Geobacillus* sp. WSUCF-018B (WP_100663940.1). Subsequently, the PW2 endoglucanase coding sequence was submitted to Genbank under accession no. MH049504. To validate the BLASTp results, phylogenetic analysis of the putative ORFs of PW1 and PW2 with their related hits was carried out. The ORFs of both PW1 and PW2 grouped with the members of M42 and M28 family peptidase of different *Geobacillus* sp. as well as endoglucanase/cellulase of thermophilic bacteria (Fig. 2c). The endoglucanase from *Bacillus* sp. PW1 formed a distinct clade, containing endoglucanase M, M42 peptidase endoglucanase, and M28 peptidase of *Geobacillus* sp., while PW2 endoglucanase coevolved with all the related members in the tree. It is rather intriguing that the PW1 and PW2 ORFs are similar to a peptidase as well as an endoglucanase, indicating the possibility for a dual enzyme activity of the putative ORFs. Therefore, in silico analysis of the two ORFs of PW1 and PW2 was undertaken to gain insights into the nature of the two proteins.

### PW1 and PW2 endoglucanases share sequence identities with representative members of thermophilic cellulase as well as peptidases

**Fig. 3:**
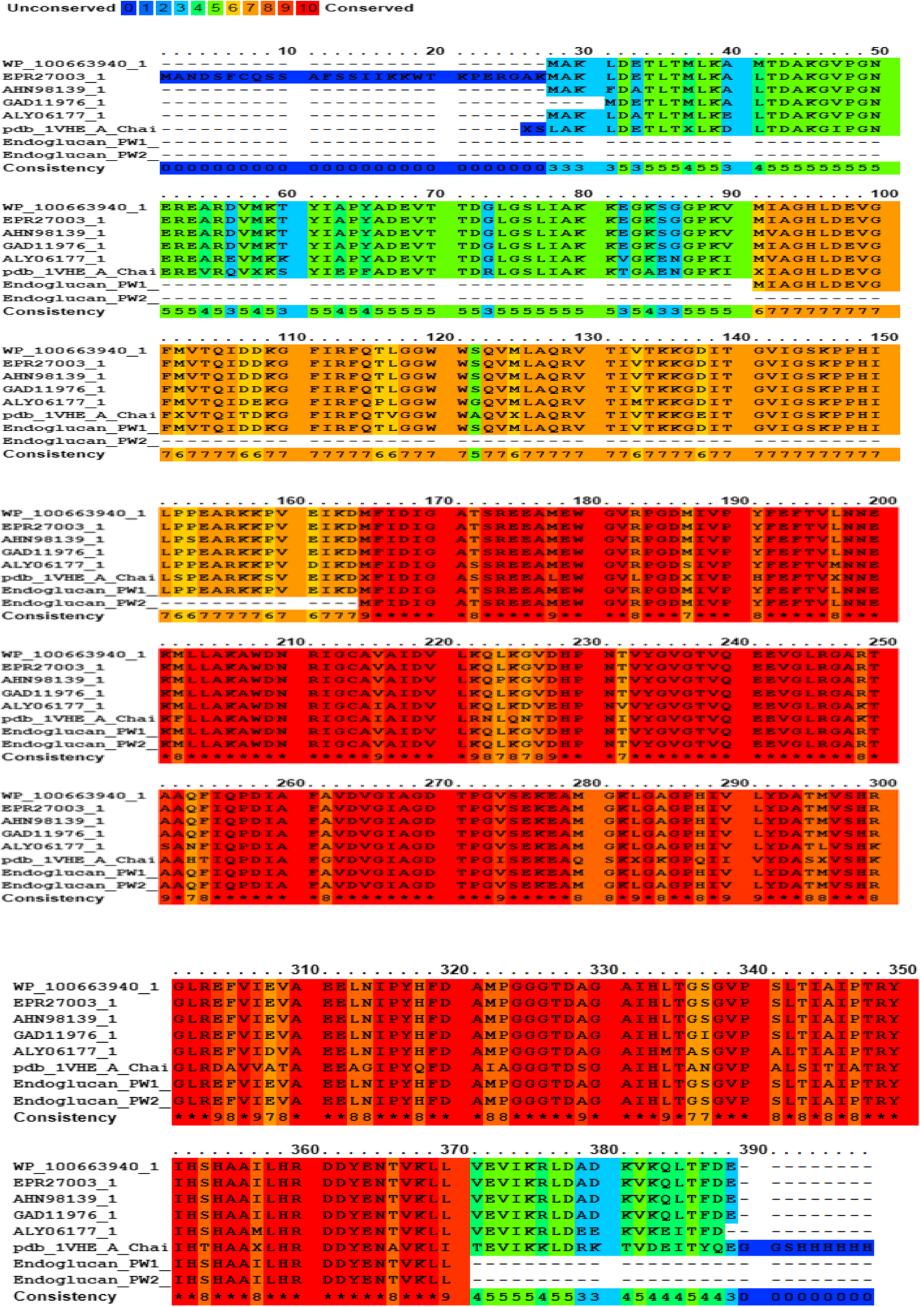
Sequence alignment of PW1 and PW2 endoglucanases with representative members of endoglucanases, cellulases, and aminopeptidase proteins. The protein sequences corresponding to M42 family peptidase of *Geobacillus* sp. WSUCF-018B (WP_100663940.1), endoglucanase M of *Geobacillus* sp. WSUCF1 (EPR27003.1), cellulase of thermophilic bacterium enrichment culture clone XM70 (AHN98139.1), cellulase of *Geobacillus kaustophilus* (GAD11976.1), cellulase of *Bacillaceae* thermophilic enrichment clone (ALY06177.1), aminopeptidase/glucanase homolog of *Bacillus subtilis* strain168 (pdb_1VHE.A Chain A), endoglucanase PW1 (*Bacillus* sp. PW1), and endoglucanase PW2 (*Bacillus* sp. PW2) were selected, and alignment was performed using PRALINE. The conservation scale is shown on the top

The protein sequences of PW1 and PW2 endoglucanases were subjected to multiple sequence alignment with related cellulase sequences as well as peptidase [M42 family peptidases and YsdC [29]. Based on the sequence analysis, PW2 endoglucanase was found to be a truncated form of PW1 endoglucanase (Fig. 3). Interestingly, both the putative ORFs of PW1 and PW2 exhibited moderate to high degree of conservation with canonical endoglucanases as well as the two amino peptidases studied (Fig. 3). These results raise the possibility that the putative ORFs encode for dual enzyme activities. Although there are not many such reports, Maiti et al. [30] reported the dual activity of a protein isolated from *Brevibacillus agri*, which exhibits gelatinase as well as cellulase activities in vitro.

### PW1 and PW2 endoglucanases possess domains of M42 peptidase/endoglucanase family

**Fig. 4:**
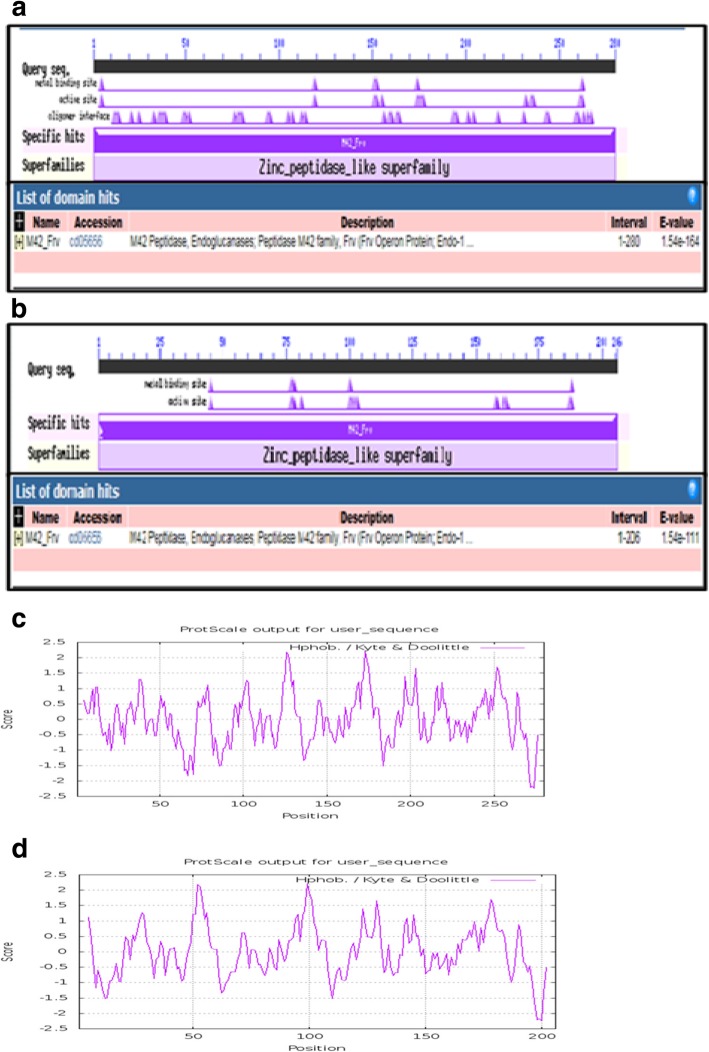
Domain analysis and hydropathy analysis of PW1 and PW2 endoglucanases. Conserved domain analyses of endoglucanase of PW1 (**a**) and PW2 (**b**). Hydropathy plots for PW1 endoglucanase protein (**c**) and PW2 endoglucanase protein (**d**) were generated using Protscale

Conserved domain analysis of PW1 and PW2 endoglucanase proteins revealed the presence of M42 peptidase/endoglucanase domain of Zinc peptidase super family in both the proteins (Fig. 4a, b). These results are consistent with the phylogenetic analysis of the putative endoglucanases of PW1 and PW2 (Fig. 2c). Further, hydropathy plots indicate that both the proteins are hydrophilic in nature with hydropathy score of − 2.4 for PW1 endoglucanase and − 2.2 for PW2 endoglucanase, respectively (Fig. 4c, d). The presence of domain with endoglucanase activity in the M42 peptidase family has been challenged by Dutoit et al. [29], wherein CelM cellulase and TmPep1050 endoglucanase were shown to be an aminopeptidase. Therefore, the identity of the putative PW1 and PW2 endoglucanase proteins needs to be validated by functional analysis of the endoglucanase and peptidase activities.

### In silico molecular docking reveals that PW1 and PW2 endoglucanases exhibit the strongest interaction with CMC, substrate of cellulase enzyme

**Fig. 5:**
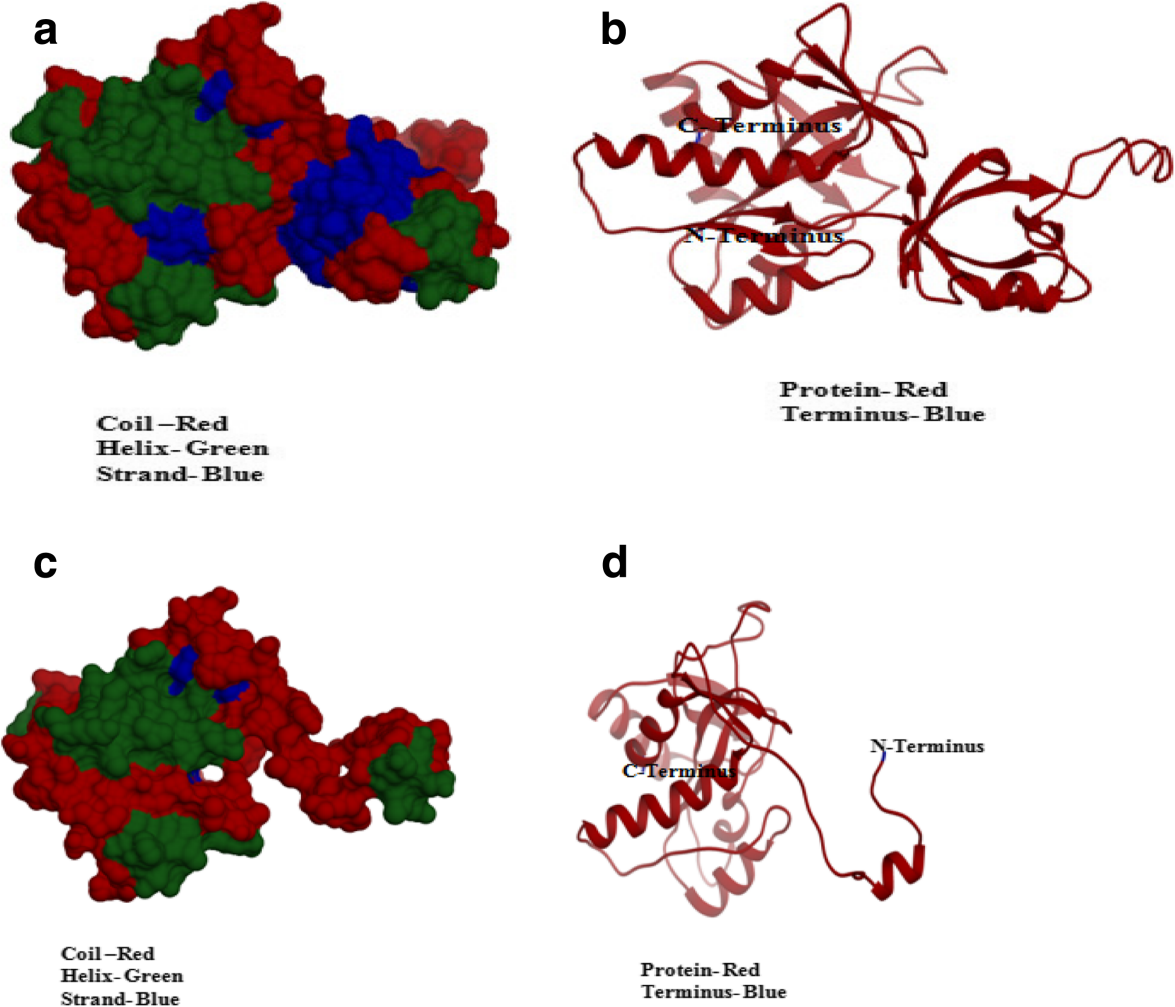
The three-dimensional molecular structures of PW1 and PW2 endoglucanases generated by homology modeling. Surface models (**a**, **c**) and ribbon models (**b**, **d**) of PW1 and PW2 endoglucanases were generated using aminopeptidase/glucanase homolog of *Bacillus subtilis* strain168 (pdb_1VHE.A) as a template. Alpha helices are colored green, beta strands are in blue, and linkers are shown in red

To gain insights into the structure of the putative endoglucanases, and their interaction with the substrates/products of cellulase, homology models were generated by the Swiss model based on the structure of 1VHE.A aminopeptidase/endoglucanase (Fig. 5). PW1 and PW2 endoglucanase sequences exhibited protein coverage of 100% and identity of 80.71% and 76.70% with the template, respectively. PW1 endoglucanase showed the presence of 32% alpha helices and 23% beta strands (Fig. 5a, b). As shown in Fig. 5c, d, PW2 endoglucanase contained 33% alpha helices and 21% beta strands. Both PW1 and PW2 endoglucanases had a large proportion (~ 50%) of the unstructured region, as indicated by the presence of linkers in the structures. Similar studies on cellulases from different *Pseudomonas* sp. indicated the predominance of random coils [31].

Subsequently, the 3D surface models were used for docking enzyme-substrate interaction with the substrates namely CMC, cellulose, xylan, and products/intermediates namely, glucose, maltose, and dextrin (Additional file 1: Figure S5 and Figure S6). The binding energy for the interactions [*E*_total_ (kcal/mol)] was calculated (Tables 2 and 3). CMC and maltose were found to be the best interacting ligands for both PW1 and PW2 endoglucanases. As expected, xylan, a hemi-cellulose, and the products like glucose and dextrin exhibited lower binding energies for both the endoglucanases. However, maltose showed strong affinity for binding, indicating an alternate binding mechanism with the enzymes. Thus, molecular docking studies favor the possible function of the putative proteins as endoglucanases, despite belonging to M42 peptidase/endoglucanase family.

**Table 2:** Binding energies (*E*_total_) for the interaction of carboxymethyl cellulose, cellulose, xylan, glucose, maltose, and dextrin with PW1 endoglucanase and the sites of interaction on the enzyme

S. no	Substrate	*E*_total_ of docking (kcal/mol)	Amino acid residues involved in interaction
1.	Carboxymethyl cellulose	− 286.36	Leu 6, Met 97, Ile98, Leu 27, Asp 7, Glu 8, Phe 11, Glu 152, Gly 10, Val 9, Val 153, Gln 150, Val 149 of chain A
2.	Cellulose	− 158.33	Ile 261, Glu 151, Asp 119, Glu 152, Val 175, Gly 236, Gly 176, Ile 177, Met 232 of chain A
3.	Xylan	− 156.39	Val 153, Gly 154, Glu 151, Leu155, Glu 152, Thr 205, Gly 236 of chain A
4.	Glucose	− 166.55	Leu 155, Gly 154, Val 153, Glu 151, Glu 152, Gly 235, Thr 205 of chain A
5.	Maltose	− 236.15	Ile 177, Ile 261, His 262, Glu 152, Gly 28, Val 153, Asp 119, Gly 154, Leu 155, Gly 235, Gly 234 of chain A
6.	Dextrin	− 171.03	Glu 8, Asp 7, Val 9, Leu 27, Met 97, Ile 98, Val 99 of chain A

**Table 3:** Binding energies (*E*_total_) for the interaction of carboxymethyl cellulose, cellulose, xylan, glucose, maltose, and dextrin with PW2 endoglucanase and the sites of interaction on the enzyme

S. no	Substrate	*E*_total_ of docking (kcal/mol)	Amino acid residues involved in interaction
1.	Carboxymethyl cellulose	− 277.10	Ile 168, Pro 93, Val 69, Ala 96, Try 70, Ile 91, Ile 95, Leu 206, Phe 97, Leu 206, Phe 97, Leu 57, Val 56, Val 72, Val 99 and Ala 53 of chain A
2.	Cellulose	− 163.43	Ser 151, Ala 152, Arg 76, Ala 153, Ser 78, Asp 66 and Leu 64 of chain A
3.	Xylan	− 154.78	Pro 93, Ile 168, Ala 88, Ile 91, Ala 87, Thr 74, Val 72 of chain A
4.	Glucose	− 169.25	Leu 64, Asp 66, Gly 62, Ser 78, Arg 76, Ala 153, Ala 152 of chain A
5.	Maltose	− 281.72	Pro 93, Ile 91, Ala96, Ile 168, Val 69, Gly 71, Leu 206, Val 52, Ala 98, Val 99 and Ala 53 of chain A
6.	Dextrin	− 175.37	Tyr 70, Leu 57, Ala 53, Gly 71, Val 56, Val 99, Phe 97, Leu 206 of chain A

## Conclusion

In the present study, we have explored two strict thermophiles of Tattapani hot spring (Himachal Pradesh, India), namely PW1 and PW2 for their cellulolytic potential. Both the bacterial strains exhibited profound thermophilic cellulase activity. The two isolates were identified as members of *Bacillus* genera by 16S rDNA sequencing, with high sequence similarities, yet distinct growth features and phylogenetic positions. The two thermophiles were mined for genes encoding cellulase activity and found to encode putative endoglucanases of a rather contentious dual family of enzymes namely M42 peptidase/endoglucanase. The putative endoglucanases of PW1 and PW2 were studied by in silico structural modeling and molecular docking with substrates of cellulases. Both of the putative proteins showed strongest binding with carboxymethyl cellulose and maltose. Biochemical studies of the putative endoglucanases in a recombinant expression system are required to validate their cellulase activities for potential industrial applications.

## Additional file

Additional file 1: Supplementary material. (DOCX 1660 kb)

## Data Availability

Supporting data is provided in the supplementary material.

## Abbreviations

CBHases: Cellobiohydrolases
CMCases: Carboxymethylcellulases
CMC: Carboxymethyl cellulose
NB: Nutrient broth
