# Supplementary material for: Endoglucanase gene of M42 aminopeptidase/endoglucanase family from thermophilic Bacillus sp. PW1 and PW2 isolated from Tattapani hot spring, Himachal Pradesh, India
Source: J Genet Eng Biotechnol. 2019 Oct 2;17:4. doi: 10.1186/s43141-019-0001-8 (PMC6821146; doi:10.1186/s43141-019-0001-8)
Supplement: Supplementary file 1 — Supplementary material. (DOCX 1660 kb) [file 43141_2019_1_MOESM1_ESM.docx]

**Supplementary material**

**Table S1.** The sequence of primers used in the study.

| **Primer** | **Sequence (5’ to 3’)** | **Nucleotides** |
| --- | --- | --- |
| 1. **27F** | AGAGTTTGATCTGGCTCG | 25 |
| 1. **1492R** | TACGGACCTTGTTACGACTT | 24 |
| 1. **F Cellulase** | ATGGCGAAGTTGGAC | 24 |
| 1. **R Cellulase** | TCAAACGTCAGTTTGT | 26 |

1. b.

**Fig S1: Effect of physical parameters (temperature and pH) on growth of microbial isolates PW1 and PW2.**Cell density of bacterial cultures was measured at 600 nm and plotted against the indicated pH (**a**) and temperature (**b**).Data of three independent experiments was plotted with standard deviation.

**a.**

**b**

**Fig S2:** Effect of carbon and nitrogen sources on growth of microbial isolates PW1 and PW2. Absorbance of bacterial cultures was measured at 600 nm and plotted against carbon sources (**a**) and nitrogen sources (**b**). Data of three independent experiments was plotted with standard deviation.

**a.**

ATGATCGCCGGCCATTTGGATGAAGTCGGCTTTATGGTGACGCAAATCGACGACAAAGGATTCATCCGCTTCCAAACGCTTGGCGGATGGTGGAGCCAAGTGATGCTCGCCCAGCGCGTGACGATCGTGACGAAAAAAGGCGACATCACCGGCGTCATCGGTTCGAAGCCGCCGCACATTCTGCCGCCGGAGGCGCGCAAAAAACCGGTGGAAATCAAAGATATGTTCATCGACATCGGCGCGACAAGCCGCGAGGAAGCGATGGAGTGGGGCGTCCGCCCGGGCGATATGATCGTGCCGTATTTTGAATTTACGGTATTGAACAATGAAAAAATGCTGCTCGCGAAAGCGTGGGACAACCGGATCGGCTGTGCGGTCGCCATCGATGTGCTCAAGCAGCTGAAAGGCGTCGACCATCCAAACACGGTATACGGCGTCGGCACGGTGCAGGAAGAAGTCGGCTTGCGCGGCGCGCGCACGGCCGCCCAATTCATTCAGCCGGATATCGCCTTTGCGGTGGATGTCGGCATTGCCGGCGATACACCGGGCGTGTCGGAAAAAGAAGCGATGGGCAAACTCGGCGCCGGCCCGCACATCGTCCTGTACGACGCGACGATGGTGTCGCACCGCGGCTTGCGCGAATTTGTCATCGAAGTGGCGGAAGAGCTGAACATTCCGTACCATTTTGATGCCATGCCGGGCGGCGGTACGGATGCGGGAGCGATTCATTTAACGGGCAGCGGCGTTCCGTCGCTCACGATCGCCATCCCGACGCGCTACATCCACTCGCACGCCGCCATTTTGCACCGCGACGACTACGAAAACACGGTCAAGCTGCTGTGA

**b.**

MIAGHLDEVGFMVTQIDDKGFIRFQTLGGWWSQVMLAQRVTIVTKKGDITGVIGSKPPHILPPEARKKPVEIKDMFIDIGATSREEAMEWGVRPGDMIVPYFEFTVLNNEKMLLAKAWDNRIGCAVAIDVLKQLKGVDHPNTVYGVGTVQEEVGLRGARTAAQFIQPDIAFAVDVGIAGDTPGVSEKEAMGKLGAGPHIVLYDATMVSHRGLREFVIEVAEELNIPYHFDAMPGGGTDAGAIHLTGSGVPSL TIAIPTRYIHSHAAILHRDDYENTVKLL

**Fig S3: a.** Nucleotide sequence of PW1 Endoglucanase gene isolated from *Bacillus* sp.PW1.**b.** Translated sequence of PW1 Endoglucanase isolated from *Bacillus* sp. PW1.

**a.** ATGTTCATCGACATCGGCGCGACAAGCCGCGAGGAAGCGATGGAGTGGGGCGTCCGCCCGGGCGATATGATCGTGCCGTATTTTGAATTTACGGTATTGAACAATGAAAAAATGCTGCTCGCGAAAGCGTGGGACAACCGGATCGGCTGTGCGGTCGCCATCGATGTGCTCAAGCAGCTGAAAGGCGTCGACCATCCAAACACGGTATACGGCGTCGGCACGGTGCAGGAAGAAGTCGGCTTGCGCGGCGCGCGCACGGCCGCCCAATTCATTCAGCCGGATATCGCCTTTGCGGTGGATGTCGGCATTGCCGGCGATACACCGGGCGTGTCGGAAAAAGAAGCGATGGGCAAACTCGGCGCCGGCCCGCACATCGTCCTGTACGACGCGACGATGGTGTCGCACCGCGGCTTGCGCGAATTTGTCATCGAAGTGGCGGAAGAGCTGAACATTCCGTACCATTTTGATGCCATGCCGGGCGGCGGTACGGATGCGGGAGCGATTCATTTAACGGGCAGCGGCGTTCCGTCGCTCACGATCGCCATCCCGACGCGCTACATCCACTCGCACGCCGCCATTTTGCACCGCGACGACTACGAAAACACGGTCAAGCTGCTGTGA

**b.**

MFIDIGATSREEAMEWGVRPGDMIVPYFEFTVLNNEKMLLAKAWDNRIGCAVAIDVLKQLKGVDHPNTVYGVGTVQEEVGLRGARTAAQFIQPDIAFAVDVGIAGDTPGVSEKEAMGKLGAGPHIVLYDATMVSHRGLREFVIEVAEELNIPYHFDAMPGGGTDAGAIHLTGSGVPSLTIAIPTRYIHSHAAILHRDDYENTVKLL

**Fig S4: a.** Nucleotide sequence of PW2 Endoglucanase isolated from *Bacillus* sp. PW2. **b.**Translated sequence of PW2 Endoglucanase isolated from *Bacillus* sp. PW2

**a.**


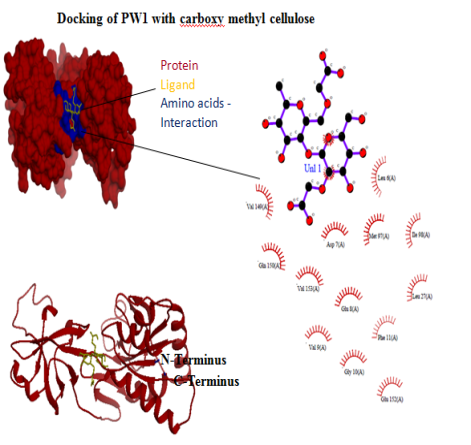


**b.**


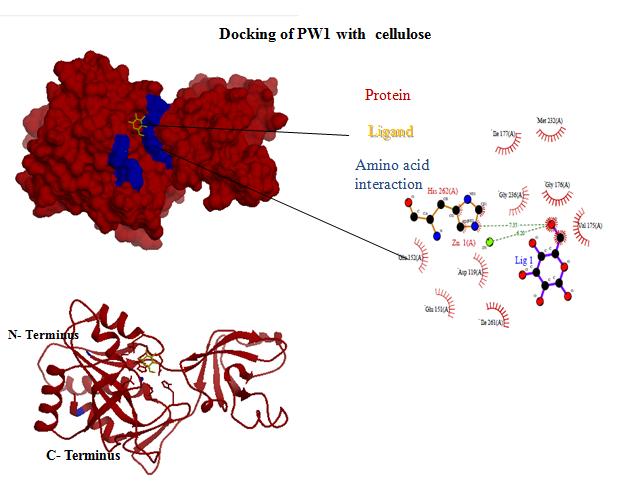


**c.**

**
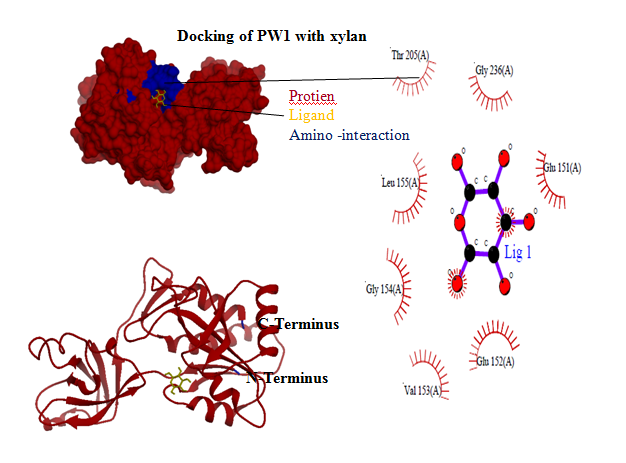
**

**d.**

**
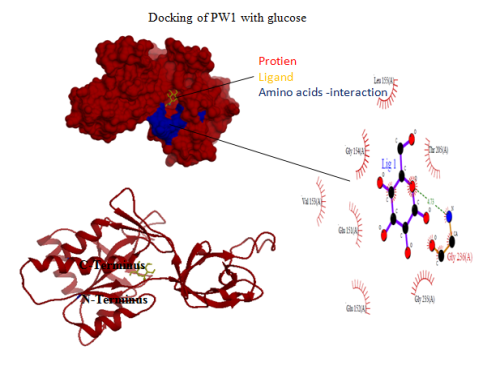
**

**e.**

**
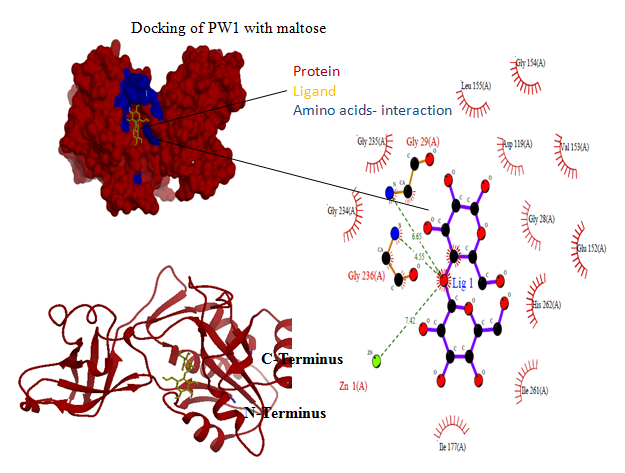
**

**f.**

**
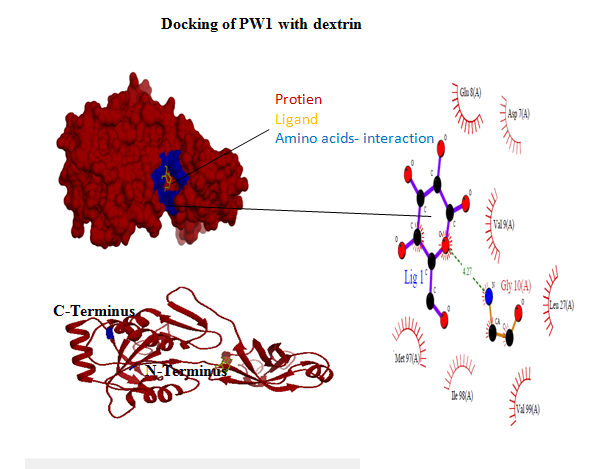
**

**Fig S5: Docking of PW1 Endoglucanase with substrates and products of cellulase.** Surface model for docking of PW1 Endoglucanase with Carboxymethyl cellulose (a), Cellulose (b), Xylan (c), Glucose (d), Maltose(e) and Dextrin(f). Each figure represents the complete surface model for docking, ribbon model for docking and panels the specific residues of PW1 Endoglucanase interacting with the respective substrates / products.

**a.**


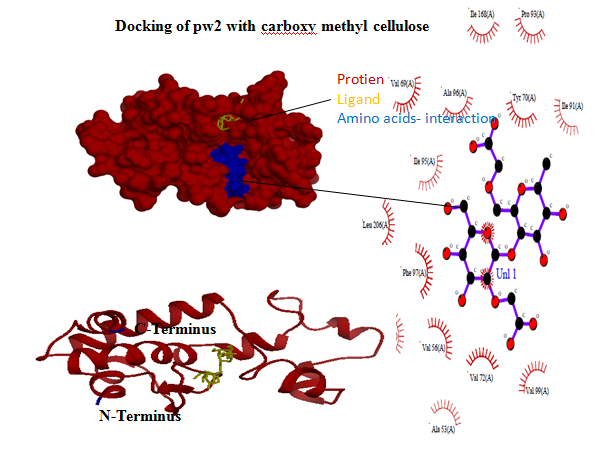


**b.**

**
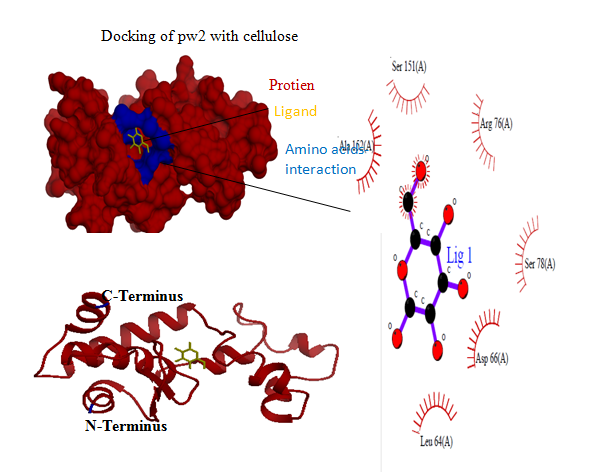
**

**c.**

**
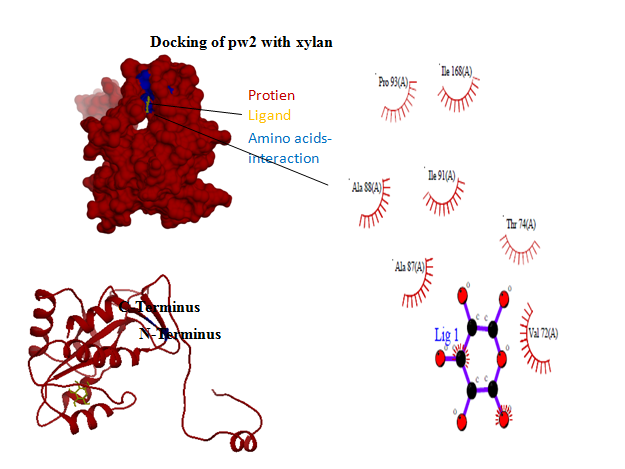
**

**d.**

**
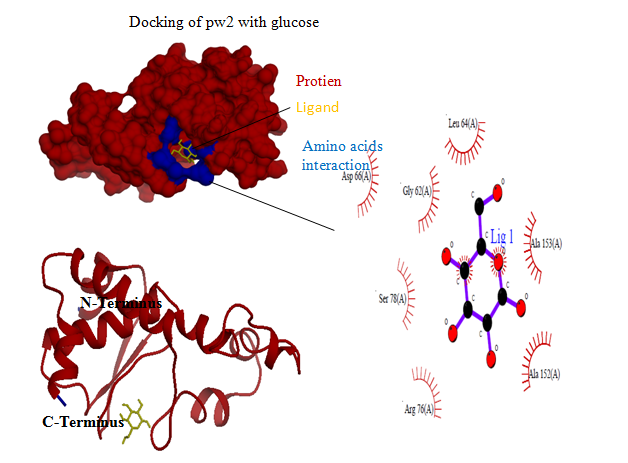
**

**e.**

**
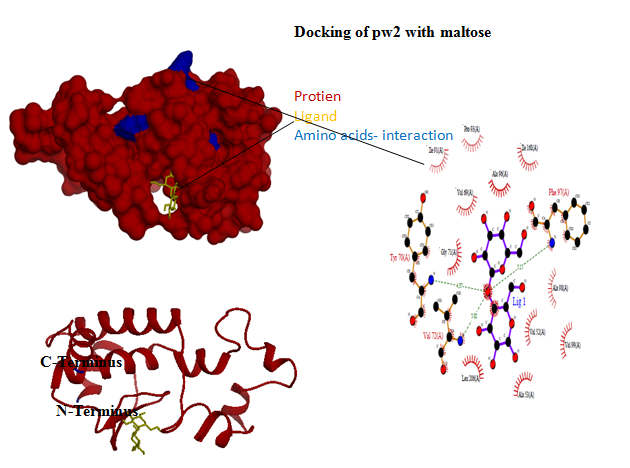
**

**f.**

**
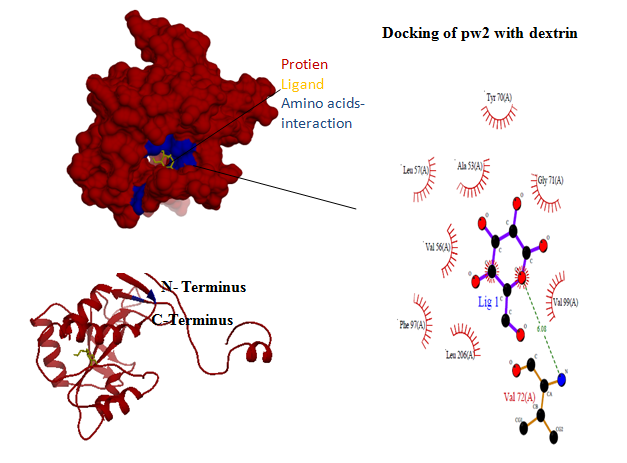
**

**Fig S6: Docking of PW2 Endoglucanase with substrates and products of cellulase.** Surface model for docking of PW2 Endoglucanase with Carboxymethyl cellulose (a), Cellulose (b), Xylan (c), Glucose (d), Maltose (e) and Dextrin (f). Each figure represents the complete surface model, ribbon model for docking and the specific residues of PW2 Endoglucanase interacting with the respective substrates / products.
